# Creole goat morphological diversity partially mirrors district-level variation in the seasonally dry forest of Piura in Peru

**DOI:** 10.1371/journal.pone.0339584

**Published:** 2025-12-31

**Authors:** José Antonio Haro-Reyes, Emmanuel Alexander Sessarego, Danny Julio Cruz, Pablo R. Gonzales-Guevara, José Antonio Ruiz-Chamorro, Juancarlos Alejandro Cruz-Luis

**Affiliations:** 1 Estación Experimental Agraria Donoso, Dirección de Servicios Estratégicos Agrarios, Instituto Nacional de Innovación Agraria, Huaral, Lima, Perú; 2 Dirección de Servicios Estratégicos Agrarios, Instituto Nacional de Innovación Agraria, Lima, Perú; 3 Facultad de Agronomía, Universidad de Buenos Aires/UBA, Ciudad Autónoma de Buenos Aires, Argentina; 4 Estación Experimental Agraria Chincha, Dirección de Servicios Estratégicos Agrarios, Instituto Nacional de Innovación Agraria, Chincha, Ica, Perú; 5 Estación Experimental Agraria El Chira, Dirección de Desarrollo Tecnológico Agrario. Instituto Nacional de Innovación Agraria (INIA), Piura, Piura, Perú; Eskisehir Osmangazi University: Eskisehir Osmangazi Universitesi, TÜRKIYE

## Abstract

Livestock systems in marginal ecosystems such as seasonally dry forests (SDFs) face increasing sustainability challenges, yet the role of morphology in mediating animal adaptation to local environmental and management conditions remains underexplored. In the Piura region of northern Peru—home to the country’s most extensive SDF and its leading hub of goat production—Creole goats represent a diverse and under-characterized resource shaped by natural and human selection. Despite Creole goats’ relevance, little is known about the spatial structure of their phenotypic variation or how it may signal emerging regional morphotypes. Addressing this gap, we conducted a comprehensive morphometric analysis of 617 female Creole goats across three distinct districts within Piura’s SDF. Using linear body measurements (LBMs), morphometric indices, and multivariate analyses, we revealed significant district-level phenotypic differentiation. Goats from Catacaos exhibited consistently larger body dimensions and higher compactness indices, forming a distinct cluster in hierarchical analyses and suggesting the emergence of a localized morphotype. Notably, this phenotypic pattern was largely driven by animals from four specific farmers, pointing to the potential influence of herd-level management practices or breeding history. Despite this within-district heterogeneity, the Catacaos subgroup remained clearly differentiated from goats in Lancones. Principal component analysis of LBMs identified a dominant size axis explaining over 70% of variance, with Catacaos goats diverging along this dimension. In contrast, morphometric indices showed weaker discriminatory power. These findings suggest that LBMs outperform derived indices in capturing fine-scale phenotypic structure and may reflect both ecological adaptation and management-driven selection. Our results underscore the potential of morphometric profiling for identifying regionally adapted livestock types and lay the groundwork for future geographic indication schemes that valorize local biodiversity and support rural livelihoods.

## 1. Introduction

The seasonally dry forest (SDF) is a semi-arid ecosystem defined by prolonged dry periods, scarce and irregular rainfall, and predominance of deciduous woody vegetation. In Peru, SDF extends across more than four million hectares, with the Piura region comprising approximately 65% of this territory [1,2]. Notably, Piura is also the country’s main center of goat production, supporting about 17% of Peru’s total caprine population, approximately 1.7 million animals nationwide [3].

Across arid and semi-arid regions worldwide, caprine production is primarily practiced under extensive, low-input systems in rural areas. These systems often represent subsistence-level livelihoods for smallholder farmers, contributing not only to food security but also providing socio-cultural and ecosystem services beyond conventional market metrics, such as income and profits [4–6]. Within these resource-limited systems, goats’ evolutionary adaptations and the pastoral traditions associated with their management make them the livestock of choice, capable of thriving where other species rarely can.

Goats exhibit a unique suite of adaptive traits—including selective browsing, drought tolerance and thermoregulation—that enable them to thrive under harsh environmental conditions [7,8], such as those characteristic of the SDF. These traits are underpinned by complex genetic adaptations shaped by both domestication and natural selection in marginal landscapes [9]. Among the various goat populations inhabiting these systems, “indigenous” or “Creole” goats are particularly valued for their rusticity and capacity to perform under resource constraints [10].

Creole goats exhibit high phenotypic and genotypic variability [5]. Morphological studies across Peruvian regions have identified multiple morphotypes and diverse phaneroptic traits, all sharing the rusticity typical of the breed and distinct adaptations for meat or milk production [11–14]. This diversity reflects the complex genetic background of Creole goats, shaped in Peru by historical crossbreeding with introduced breeds such as Saanen, Alpine, Oberhasli, Toggenburg, and predominantly Anglonubian [15]. However, genome-wide analyses using single nucleotide polymorphisms (SNPs) have confirmed substantial genetic divergence from these ancestral breeds, as well as notable differentiation among Creole populations throughout the Americas [16]. Particularly, Northern Peruvian creole goats display high genetic diversity and low inbreeding coefficients [17], suggesting localized adaptation and the potential emergence of distinct regional morphotypes.

Morphometric traits, including linear body measurements and derived indices, are often heritable and closely associated with functional performance. They represent a robust and low-cost approach for estimating body weight, characterizing phenotypic diversity, identifying breeds, and designing targeted selection programs, particularly in resource-limited production systems [18–20]. Beyond their practical utility, morphometric profiles can contribute together with other features as distinguishing elements for regional-types differentiation and valorization. For instance, although Chile’s Chilote sheep are genetically similar to local Spanish breeds, their product quality distinctiveness, geographic origin, and consumer preference supported their recognition as a local breed, together with a characteristic morphology [21–23]. Similarly, the Italian geographical indication “Vitellone Bianco dell’Appennino Centrale” encompasses multiple local cattle breeds, each contributing specific traits that justify collective branding [24].

Indeed, in the European Union and beyond, geographical origin distinction has become a key component in the marketing of traditional livestock products. Such certifications are associated with increased consumer willingness to pay and contribute to the rural development [25]. In this context, livestock morphology transcends mere form—it functions as a proxy for local adaptation, a tool for regional typification, and a lever for economic valorization of animal products [26].

Given Piura’s ecological diversity and its importance to national goat production, it is plausible that distinct Creole goat morphotypes have emerged across districts, shaped by the local landscapes, management practices, and microclimates. However, previous research has largely examined morphometric variation at regional scales, without addressing ecosystem-level patterns that may more directly capture the influence of local natural resources and physiographic heterogeneity. In this study, we assess whether morphometric variation among Creole goat populations reflects district-level conditions within the SDF. This research aims to support the identification of regionally adapted morphotypes and provide a foundation for their future recognition in conservation and inclusion in geographic indications.

## 2. Materials and methods

### 2.1. Study area

**Fig 1:**
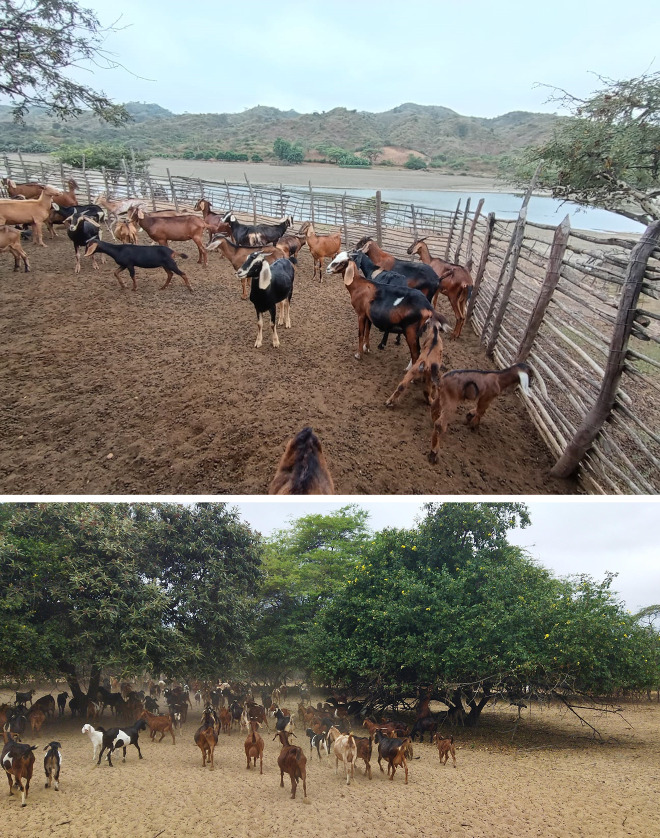
Typical Creole goats and grazing landscape in Piura region, Peru.

Creole goat herds (Fig 1) were sampled from representative districts within the Piura region of northern Peru, where caprine production is predominantly meat oriented [12]. The selected districts—Catacaos, Chulucanas, and Lancones—are located in the provinces of Paita, Morropón, and Sechura, respectively. According to the Peruvian National Ecosystem Map, Catacaos is classified within the Plains SDF (Bosque Estacionalmente Seco – llanura; BES-ll) sub-ecosystem, Lancones is situated within the Hill and Mountain SDF (Bosque Estacionalmente Seco – colinas y montañas; BES-cm) sub-ecosystem (Table 1) [1]. Catacaos and Lancones comprise the largest district areas within their respective SDF ecosystem, whereas Chulucanas spans a transitional zone encompassing both ecosystems. Collectively, the three districts cover more than 20% of each ecosystem and harbor roughly one-third of the goat population in the Piura region.

**Table 1:** Surface area of intersections of SDF ecosystems and districts in Piura region.

District	Ecosystem	Surface area (ha)	Surface (%) relative to the ecosystem within Piura
Catacaos	Plains SDF	210473.06	22.15
Chulucanas	Plains SDF	37162.41	3.91
Chulucanas	Hills and Mountains SDF	17893.24	1.65
Lancones	Hills and Mountains SDF	206688.67	19.02
**TOTAL**		**472217.38**	

Both SDF ecosystems are deciduous and semi-arid, characterized by low and highly variable seasonal rainfall. The Hills and Mountains SDF is defined by dissected terrain with local reliefs ranging from 30 to 300 m above the surrounding plains and slopes of 15–80%, in sharp contrast to the flat topography of the Plains SDF [27]. The two sub-ecosystems also differ in floristic composition [28], which influences forage availability and intake, as all animals graze on native rangelands in an extensive system without supplementary feeding. The Hill and Mountain SDF landscape supports denser, arboreal vegetation, while the Plains SDF are more open. In addition, herds in Catacaos have seasonal access to the agricultural valley of Bajo Piura, where animals forage on post-harvest crop residues providing an alternative and nutritionally distinct feed resource during the dry season.Principio del formulario

Final del formulario

### 2.2. Ethical statement

Ethics committee approval was not required for this study, as no experimental procedures or invasive interventions were conducted. Morphometric measurements were collected non-invasively during routine animal handling, causing no additional distress. All animal management and handling were conducted by the owners themselves as part of standard husbandry practices, in accordance with Peruvian National Law No. 30407 on Animal Protection and Welfare. Oral informed consent was obtained from all herd owners prior to data collection. The study was conducted as part of the PROCAP project (CUI 2506684), with authorization from the Dirección de Servicios Estratégicos Agrarios (DSEA) of the Instituto Nacional de Innovación Agraria (INIA), the national authority on agricultural innovation in Peru.

### 2.3. Sampling procedures

The study included 617 female goats sampled from 34 herds between September and November 2023. Only females were selected to minimize variability associated with sex. Age was estimated based on dental eruption patterns and classified into two categories: 1–2 years (eruption of 2–4 permanent incisors) and ≥3 years (eruption of six or more permanent incisors). The age distribution was balanced across the three sampled districts.

**Fig 2:**
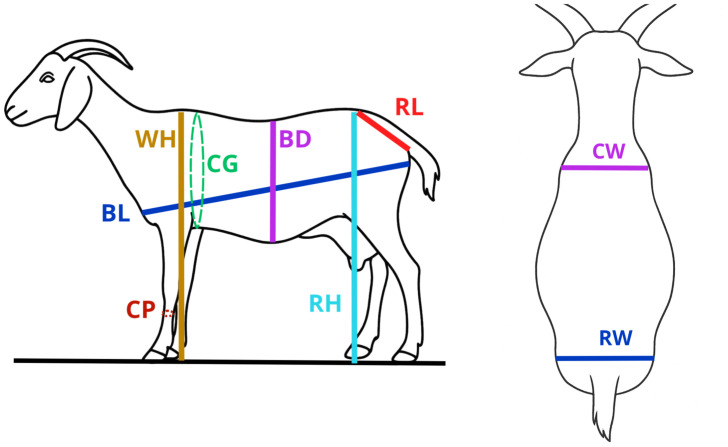
Linear body measurements taken from Piura goats. WH: withers height; RH: rump height; BL: body length; CG: chest girth; CP: cannon perimeter; RL: rump length; BD: body depth; CW: chest width, RW: rump width.

All animals were managed under traditional extensive production systems. Goats were housed in group pens overnight and allowed to graze freely during the day on the SDF rangelands, without supplemental feeding. Body weight (BW) and linear body measurements (LBMs) (Fig 2, Table 2) were recorded in the early morning, prior to grazing, to avoid postprandial variation. BW was measured using a portable hook-type scale, while LBMs were obtained using a flexible measuring tape and a measuring stick. Based on these traits, ten morphometric indices were calculated (Table 3) to describe body conformation and its association with productive potential. The herds were distributed across the three study districts, which together account for over 30% of the goat population in the Piura region [29], and the number of animals sampled per district is shown in Table 4.

**Table 2:** Descriptions for assessment of body weight and linear body measurements.

Morphometric traits	Acronym	Measurement descriptions
Body weight	BW	Assessed using a digital hanging scale, ensuring minimal animal movement for accuracy.
Withers height	WH	Vertical distance from the dorsal point between the scapulae to the ground.
Rump height	RH	Vertical measurement from the highest point of the rump to the ground surface.
Body length	BL	Linear distance from the greater tubercle of the humerus to the ischial tuberosity, following the body axis.
Chest girth	CG	Circumference measured immediately posterior to the scapulae, encircling the thoracic cavity perpendicular to the body’s longitudinal axis.
Cannon perimeter	CP	Circumferential measurement at the midpoint of the metacarpal region, just distal to the carpal joint.
Rump length	RL	Distance between the anterior prominence of the ilium (hook bone) and the posterior prominence of the ischium (pin bone).
Body depth (abdominal)	BD	Vertical distance from the dorsal midline to the lowest point of the abdomen.
Chest width	CW	Horizontal width between the outermost points of the shoulder joints, just caudal to the forelimbs.
Rump width	RW	Linear distance between the lateral extremities of the tuber coxae (hip bones).

**Table 3:** Determination of morphometric indices from LBM.

Morphometric indices	Acronym	Calculation formulas* (x 100)	Body types	Reference
Body Index	BOI	Body length/ Chest girth	Longilinear: > 0.90 Medilinear: 0.86 - 0.88 Brevilinear: < 0.85	[31]
Pelvic Index	PVI	Rump width/ Rump length	Convexilinear < 100	[32]
Proportionality Index	PPI	Withers height/ Body length	Short-bodied: < 95 Medium-bodied: 95–105 Tall bodied: > 105	[13]
Metacarpal-thoracic index	MTI	Canon perimeter/ Chest girth	Hypermeter: > 11 (Meat breed) Eumeter: > 10 and <11 Ellipometric: < 10	[32]
Transversal pelvic index	TPI	Rump width/ Withers height	Meat-type animal: > 33	[33]
Longitudinal pelvic index	LPI	Rump length/ Rump height	Meat-type animal: > 37	[33]
Compactness index**	CCI	Body weight/ Withers height	Meat-type animals: > 315% Dual-purpose animals: ~ 275% Milk-type animals: ~ 260%	[34]
Relative Cannon Thickness Index	RCTI	Canon perimeter/ Withers height		
Cannon Load Index	CLI	Canon perimeter/ Body weight		

* Longitudinal measurements in cm, weight in Kg.

** Index values for body type classification were adapted from references.

**Table 4:** Descriptive statistics of body weight and linear body measurements in goats from three districts of Piura.

District	n	BW			WH			RH			BL			CG			CP			RL			BD			CW			RW		
		Mean (Kg)	± SD (Kg)	CV (%)	Mean (cm)	± SD (cm)	CV (%)	Mean (cm)	± SD (cm)	CV (%)	Mean (cm)	± SD (cm)	CV (%)	Mean (cm)	± SD (cm)	CV (%)	Mean (cm)	± SD (cm)	CV (%)	Mean (cm)	± SD (cm)	CV (%)	Mean (cm)	± SD (cm)	CV (%)	Mean (cm)	± SD (cm)	CV (%)	Mean (cm)	± SD (cm)	CV (%)
Catacaos	180	49.36	10.22	20.72	71.85	4.48	6.24	73.48	4.99	6.80	62.89	4.59	7.31	86.17	6.44	7.47	9.32	0.73	7.83	24.12	2.14	8.88	33.94	2.57	7.57	19.12	2.53	13.24	18.64	1.91	10.22
Chulucanas	299	41.93	8.71	20.76	65.02	5.05	7.77	66.76	4.87	7.30	58.22	4.72	8.11	78.00	5.97	7.65	8.10	0.68	8.43	21.48	1.93	8.99	29.77	2.46	8.27	16.40	2.00	12.18	16.67	2.18	13.09
Lancones	138	37.27	7.50	20.11	65.32	4.74	7.25	66.01	4.53	6.87	58.36	4.79	8.21	76.89	5.99	7.79	8.02	0.84	10.49	21.35	1.77	8.31	30.78	2.47	8.01	15.61	2.10	13.45	16.66	1.56	9.35
Total	617	43.06	9.96	23.14	67.08	5.71	8.51	68.56	5.78	8.43	59.61	5.15	8.63	80.14	7.24	9.04	8.44	0.93	10.99	22.22	2.31	10.39	31.21	3.07	9.84	17.02	2.59	15.21	17.24	2.17	12.58

n: number of animals. SD: standard deviation. CV: coefficient of variation. BW: body weight; WH: withers height; RH: rump height; BL: body length; CG: chest girth; CP: cannon perimeter; RL: rump length; BD: body depth; CW: chest width, RW: rump width

Descriptions based on references [30].

### 2.4. Data analysis

All statistical analyses were conducted in R software (version 4.5.0) [35], using packages from the R ecosystem unless otherwise specified. Descriptive statistics (mean, standard deviation, and coefficient of variation) were calculated for BW, LBMs, and morphometric indices, stratified by district. These metrics were used to evaluate within-group variability and to guide the selection of subsequent multivariate analyses. To explore the underlying phenotypic structure, we applied a sequence of multivariate techniques: hierarchical clustering analysis (HCA), principal component analysis (PCA), Random Forest (RF) classification, and permutational multivariate analysis of variance (PERMANOVA).

We first used HCA to assess phenotypic similarity among individuals and compare the clusters with the distribution of animals among districts. The analysis was based on Euclidean distances and Ward’s minimum variance method. Clustering was implemented with the *cluster* package, and cluster structure was visualized and evaluated using *factoextra* [36]. Cluster validity was quantified using the cophenetic correlation coefficient.

PCA was then performed to reduce dimensionality and identify major axes of morphometric variation. Two separate PCA models were performed: one based on BW and LBMs, and another based on morphometric indices. Prior to analysis, all variables were standardized (mean = 0, standard deviation = 1). PCA was performed using the *FactoMineR* package [37], and visualization was carried out with *factoextra*.

To identify the morphometric traits that most contributed to inter-district morphological differentiation—and thereby facilitate the future recognition of distinct biotypes—we implemented a Random Forest (RF) classification model using the *randomForest* package. [38]. RF is a nonparametric ensemble method, robust to multicollinearity and capable of modeling nonlinear relationships without requiring assumptions of normality or homogeneity of variances, making it appropriate for the characteristics of our dataset. The algorithm builds multiple decision trees from bootstrap samples of the original data, where at each node a random subset of predictors is tested to identify the variable and threshold that most effectively increase node purity [39]. The optimal number of trees was defined based on the stabilization of the out-of-bag (OOB) error estimate (S Fig 1). Variable importance was quantified by the mean decrease in the Gini index, which reflects the average reduction in node impurity when a variable is used for splitting across all trees. Higher values indicate stronger discriminatory power of the variable.

Finally, PERMANOVA was used to test for differences in overall morphometric parameters among districts. Two models were fitted: one for LBMs and one for morphometric indices, both based on Euclidean distance matrices. The analysis was implemented using the *adonis2* function from the *vegan* package [40]. Post hoc comparisons among districts were performed using the *pairwiseAdonis* function. Morphometric trait-specific differences between districts were evaluated using the non-parametric Wilcoxon test with Benjamini–Hochberg correction. Statistical significance was defined as *p* < 0.05.

## 3. Results

### 3.1. Descriptive statistics

The number of sample animals varied across districts, with Chulucanas contributing the largest proportion, followed by Catacaos and Lancones. BW was the most variable trait, exhibiting a coefficient of variation (CV) exceeding 20%, considerably higher than that observed for other LBMs. Among morphometric indices, calculated from LBMs as described in Table 3, the CCI and CLI showed the greatest variability, both surpassing 16% CV (Tables 4 and 5). Correlation matrices of the morphometric traits were also calculated and presented in S Table 1 and S Table 2 in S2 File.

**Table 5:** Descriptive statistics of morphometric indices in goats from three districts of Piura.

District	n	BOI			PVI			PPI			MTI			TPI			LPI			CCI			RCTI			CLI		
		Mean	± SD	CV (%)	Mean	± SD	CV (%)	Mean	± SD	CV (%)	Mean	± SD	CV (%)	Mean	± SD	CV (%)	Mean	± SD	CV (%)	Mean	± SD	CV (%)	Mean	± SD	CV (%)	Mean	± SD	CV (%)
Catacaos	180	73.19	5.28	7.21	77.47	6.71	8.66	114.55	7.23	6.31	10.84	0.71	6.58	25.95	2.17	8.38	32.86	2.36	7.18	68.44	12.39	18.11	12.98	0.84	6.45	19.50	3.16	16.21
Chulucanas	299	74.75	4.62	6.19	77.55	6.91	8.91	111.99	7.73	6.90	10.40	0.62	5.99	25.63	2.63	10.27	32.22	2.44	7.59	64.20	10.77	16.77	12.49	1.00	7.98	19.92	3.18	15.95
Lancones	138	76.04	5.25	6.90	78.39	8.30	10.59	112.36	8.80	7.84	10.44	0.82	7.86	25.58	2.53	9.90	32.41	2.63	8.12	56.91	9.81	17.24	12.31	1.26	10.28	22.09	3.42	15.47
Total	617	74.59	5.06	6.79	77.71	7.18	9.24	112.82	7.91	7.01	10.54	0.72	6.87	25.71	2.49	9.67	32.45	2.48	7.63	63.81	11.79	18.49	12.59	1.05	8.36	20.28	3.37	16.62

*n*: number of animals. SD: standard deviation. CV: coefficient of variation. BOI: body index; PVI: pelvic index; PPI: proportionality index; MTI: metacarpal-thoracic index; TPI: transversal pelvic index; LPI: longitudinal pelvic index; CCI; compactness index; RCTI: relative cannon thickness index; CLI: cannon load index.

### 3.2. Hierarchical clustering of linear body measurements groups majority of Catacaos Creole goats

**Fig 3:**
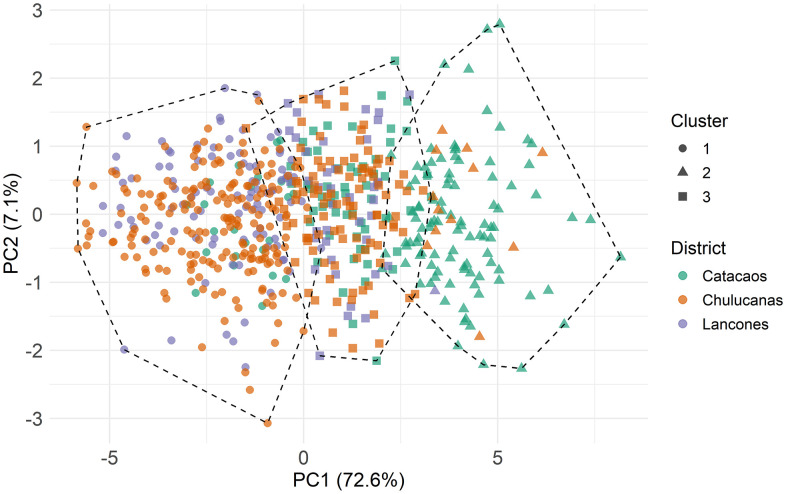
Hierarchical clustering analysis for body weight and linear body measurements.

Hierarchical clustering analysis of LBMs yielded a cophenetic correlation coefficient of 0.581, indicating that the grouping moderately reflected the morphometric distances among individuals. Imposing a three-cluster solution revealed a distinct grouping of Catacaos animals in Cluster 2 (Fig 3), which comprised 88.6% Catacaos individuals and represented 56.1% of all goats from this district (Tables 6 and 7). In contrast, Lancones goats were scarcely represented in this cluster, representing only 0.9% of the individuals assigned to Cluster 2 and 0.7% of the total Lancones sample Tables 6 and 7.

**Table 6:** Distribution of animals (%) classified by district within hierarchical clusters after LBMs analysis.

Cluster	Catacaos	Chulucanas	Lancones	Total
**1**	7.5	61.4	31.2	100.0
**2**	88.6	10.5	0.9	100.0
**3**	28.7	50.3	21.0	100.0

**Table 7:** Distribution of animals (%) classified by hierarchical clusters within districts after LBMs analysis.

Cluster	Catacaos	Chulucanas	Lancones
**1**	12.8	63.2	69.6
**2**	56.1	4.0	0.7
**3**	31.1	32.8	29.7
**Total**	100.0	100.0	100.0

In contrast, Clusters 1 and 3 contained mixed representations from Chulucanas and Lancones. Cluster 1 included 69.6% of Lancones individuals, although Chulucanas goats predominated overall, with Catacaos animals being least represented. When clustering patterns were mapped by farmer, four Catacaos herds (ASV, HZY, JLBV, and MAV) contributed over 50% of their goats to Cluster 2. All Lancones farmers contributed more than 60% of their animals to Cluster 1 (s 3 and 4).

**Fig 4:**
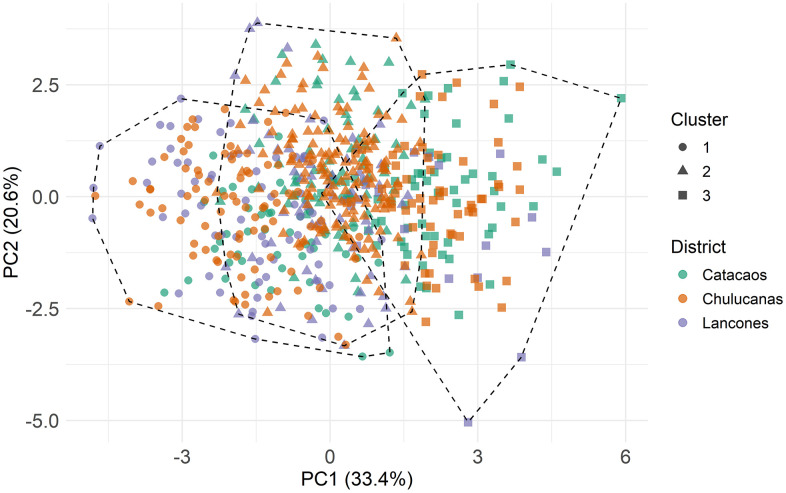
Hierarchical clustering analysis for morphometric indices.

In contrast, hierarchical clustering based on morphometric indices revealed no clear district-level separation. All clusters showed substantial overlap, with individuals from all three regions distributed throughout (Fig 4).

### 3.3. Principal component analysis reveals high contribution of variables in the first component

**Fig 5:**
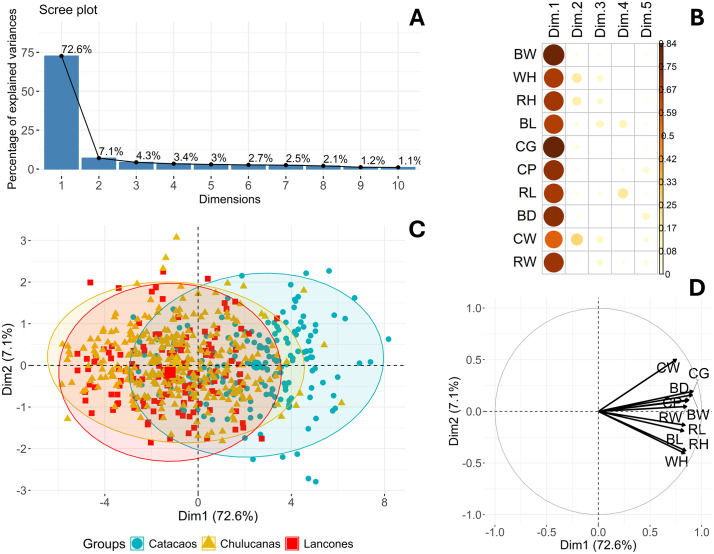
Principal component analysis of body weight and linear body measurements of Creole Goats from Piura. A: Scree plot showing percentage of explained variances per dimension (Principal Component); B: Correlation plot of cos2 of variables with all dimensions of the PCA; C: PCA scatterplot grouping data by district; D: PCA variable correlation plot.

PCA of the body weight and 9 LBMs revealed a strong underlying structure, with the first principal component (Dim1) accounting for 72.6% of the total variance (Figs 5A and 5C). Dim1 displayed high positive loadings for BW, CG, CP, and BD, with the remaining variables also contributing moderately (Figs 5B and 5D; S Table 5 in S2 File). The Kaiser–Meyer–Olkin (KMO) measure of 0.941 confirmed sampling adequacy, and Bartlett’s test (*p* < 0.001) indicated that inter-variable correlations significantly departed from an identity matrix, demonstrating that the dataset contained sufficient shared variance to justify dimensionality reduction.

**Fig 6:**
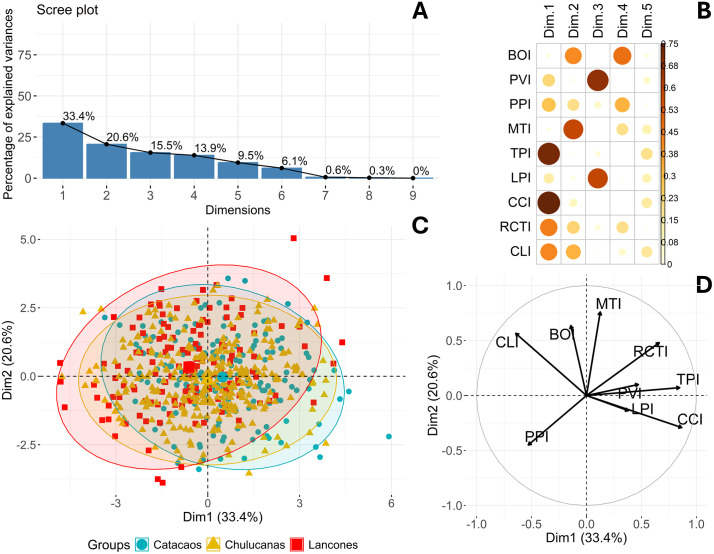
Principal component analysis for morphometric indices of Creole Goats from Piura. A: Scree plot showing percentage of explained variances per dimension (Principal Component); B: Correlation plot of cos2 of variables with all dimensions of the PCA; C: PCA scatterplot grouping data by district; D: PCA variable correlation plot.

PCA plots showed partial spatial separation of goats from Catacaos compared to those from Chulucanas and Lancones, although considerable overlap persisted. For morphometric indices, PCA identified three dimensions which together explained about 70% of the cumulative variance (Fig 6A). Dim1, Dim2 and Dim3 explained 33.4%, 20.6% and 15.5% of the variance, respectively. Dim1 was characterized by high loadings from TPI and CCI. Dim2 was driven primarily by MTI and BOI loadings, while Dim3 mainly by PVI and LPI (Fig 6B; S Table 6 in S2 File). All contributions were positive (Fig 6D). When visualized across districts, PCA showed broad overlap of animals when morphometric indices were evaluated, although Catacaos and Chulucanas appeared more similar along the first dimension (Fig 6C).

### 3.4. District-level differences in linear body measurements and morphometric indices

Descriptive comparisons of LBMs by district (Table 4) revealed only subtle morphological differences. However, multivariate analysis via PERMANOVA detected significant variation in morphometry by LBMs among the three districts (S Table 7a in S2 File).

Goats from Catacaos consistently exhibited larger body dimensions (*p* < 0.05), including BW, WH, RH, BL, CG, CP, RL, BD, CW, and RW, relative to the other districts (Table 8). In contrast, only a few parameters differed significantly between Chulucanas and Lancones, with Chulucanas goats showing greater BW and CW values, whereas BD was greater in Lancones.

**Table 8:** Pairwise comparison of individual LBMs and body weight between districts.

Morphometric trait	Comparison	Estimate	W statistic	Adjusted p-value
BW	Catacaos vs Chulucanas	7.40	38048	<0.001
	Catacaos vs Lancones	12.00	20582	<0.001
	Chulucanas vs Lancones	4.60	27072	<0.001
WH	Catacaos vs Chulucanas	7.00	45186	<0.001
	Catacaos vs Lancones	7.00	20854	<0.001
	Chulucanas vs Lancones	−0.50	19674	0.435
RH	Catacaos vs Chulucanas	7.00	44692	<0.001
	Catacaos vs Lancones	7.50	21454	<0.001
	Chulucanas vs Lancones	0.50	22098	0.231
BL	Catacaos vs Chulucanas	5.00	41396	<0.001
	Catacaos vs Lancones	5.00	18897	<0.001
	Chulucanas vs Lancones	−9.3 x 10^−5^	20006	0.610
CG	Catacaos vs Chulucanas	8.00	44614	<0.001
	Catacaos vs Lancones	10.00	21304	<0.001
	Chulucanas vs Lancones	1.00	22931	0.061
CP	Catacaos vs Chulucanas	1.00	47061	<0.001
	Catacaos vs Lancones	1.00	21528	<0.001
	Chulucanas vs Lancones	5.3 x 10^−5^	21536	0.445
RL	Catacaos vs Chulucanas	3.00	44442	0.006
	Catacaos vs Lancones	3.00	20954	<0.001
	Chulucanas vs Lancones	4.5 x 10^−5^	21460	0.494
BD	Catacaos vs Chulucanas	4.00	47106	<0.001
	Catacaos vs Lancones	3.00	20069	<0.001
	Chulucanas vs Lancones	−1.00	15795	<0.001
CW	Catacaos vs Chulucanas	3.00	43789	<0.001
	Catacaos vs Lancones	3.50	21514	<0.001
	Chulucanas vs Lancones	1.00	25298	<0.001
RW	Catacaos vs Chulucanas	2.00	40262	<0.001
	Catacaos vs Lancones	2.00	19611	<0.001
	Chulucanas vs Lancones	2.9 x 10^−5^	21278	0.594

BW: body weight; WH: withers height; RH: rump height; BL: body length; CG: chest girth; CP: cannon perimeter; RL: rump length; BD: body depth; CW: chest width, RW: rump width. Pairwise Wilcoxon test with Benjamini–Hochberg adjustment; adjusted *p* < 0.05 considered significant.

A similar trend emerged in morphometric indices, based on both descriptive statistics (Table 5) and PERMANOVA results (S Table 7b in S2 File). Catacaos goats exhibited higher values (*p* < 0.05) for BOI, PPI, MTI, CCI, and RCTI (Table 9). Chulucanas goats showed higher BOI and CCI compared to Lancones. Significant differences in LPI were observed only between Catacaos and Lancones.

**Table 9:** Pairwise comparison of individual morphometric indices between districts.

Morphometric indices	Comparison	Estimate	W statistic	Adjusted p-value
BOI	Catacaos vs Chulucanas	−1.40	22250	0.002
	Catacaos vs Lancones	−2.60	8821	<0.001
	Chulucanas vs Lancones	−1.10	18032	0.034
PVI	Catacaos vs Chulucanas	−0.80	25282	0.714
	Catacaos vs Lancones	−0.30	12003	0.714
	Chulucanas vs Lancones	9.8 x 10^−5^	21082	0.714
PPI	Catacaos vs Chulucanas	2.60	32238	0.001
	Catacaos vs Lancones	2.30	14392	0.023
	Chulucanas vs Lancones	−0.30	20214	0.735
MTI	Catacaos vs Chulucanas	0.40	36680	0.780
	Catacaos vs Lancones	0.40	16160	<0.001
	Chulucanas vs Lancones	−2.9 x 10^−5^	20198	0.724
TPI	Catacaos vs Chulucanas	0.20	27812	0.539
	Catacaos vs Lancones	0.40	13877	0.219
	Chulucanas vs Lancones	0.30	21930	0.435
LPI	Catacaos vs Chulucanas	0.70	31566	0.005
	Catacaos vs Lancones	0.50	13881	0.108
	Chulucanas vs Lancones	−0.20	19559	0.382
CCI	Catacaos vs Chulucanas	3.90	31990	0.001
	Catacaos vs Lancones	11.20	19090	<0.001
	Chulucanas vs Lancones	7.40	28556	<0.001
RCTI	Catacaos vs Chulucanas	0.50	35608	<0.001
	Catacaos vs Lancones	0.60	16763	<0.001
	Chulucanas vs Lancones	0.10	21855	0.318
CLI	Catacaos vs Chulucanas	−0.40	25112	0.220
	Catacaos vs Lancones	−2.60	7134	<0.001
	Chulucanas vs Lancones	−2.20	13126	<0.001

BOI: body index; PVI: pelvic index; PPI: proportionality index; MTI: metacarpal-thoracic index; TPI: transversal pelvic index; LPI: longitudinal pelvic index; CCI; compactness index; RCTI: relative cannon thickness index; CLI: cannon load index. Pairwise Wilcoxon test with Benjamini–Hochberg adjustment; adjusted *p* < 0.05 considered significant.

### 3.5. Body depth, body weight, and cannon perimeter are key traits distinguishing goats by district

**Fig 7:**
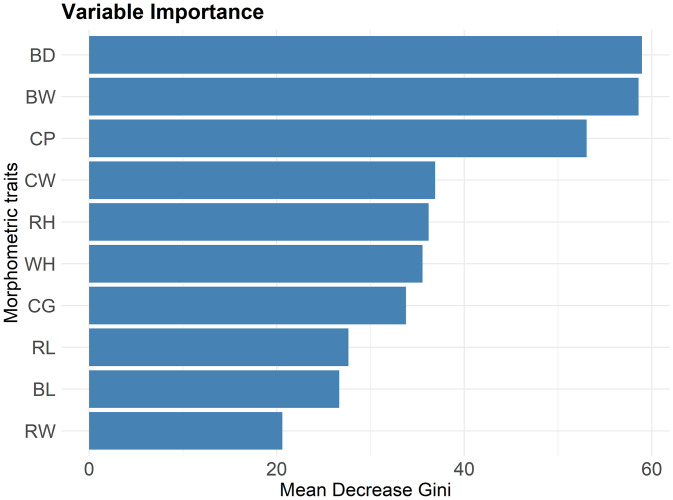
Random forest variable importance plot of body weight and LBMs classifying goats by district.

The contribution of individual morphological traits to district-level differentiation was assessed using RF analysis. Although all LBMs contributed meaningfully to the model, three traits—BD, BW, and CP—emerged as the most discriminative (S Table 8 in S2 File; Fig 7), showed the highest discriminative power (S Table 8 in S2 File; Fig 7), highlighting their relevance in distinguishing morphotypes among districts. The remaining variables exhibited moderate importance.

## 4. Discussion

Efforts to characterize the morphological diversity of the Peruvian Creole goat remain limited, although recent studies have documented marked phenotypic variation across regions. Populations inhabiting the northern dry forests exemplify this diversity, showing broad variability in body conformation and coat color [12], which complicates differentiation based solely on external appearance. Despite such heterogeneity, certain traits appear conserved across local populations. Features related to body proportionality and the predominance of brevilinear types with convex pelvic lines suggest potential suitability for meat production in diverse environments [12–14]. Nevertheless, subtle yet consistent morphometric differences within regions point to the existence of distinct local morphotypes [14], underscoring the value of LBMs as discriminating variables.

This study provides a comprehensive assessment of morphometric variation in goats from three districts within the Piura region, a key caprine production zone encompassing diverse SDF ecosystems. Multivariate analyses revealed subtle but consistent group distinctions that only partially aligned with geographic location. In contrast, analyses of individual morphometric traits showed clear differentiation among districts. Together, these patterns suggest that additional sources of variation—potentially related to differences in husbandry practices or crossbreeding among farmers—may underlie the observed morphometric structure.

Hierarchical clustering underscored the distinctiveness of Catacaos population, though moderate cophenetic correlation coefficient indicates that while the dataset is moderately structured, substantial morphological overlap persists among districts. Such overlap is expected given their geographic proximity and participation in shared markets, which facilitates genetic exchange and outbreeding. Cluster analysis based on LBMs identified a discrete group (Cluster 2) composed predominantly of Catacaos goats, representing over half of the district’s individuals, and containing almost no animals from Lancones, revealing clear morphological divergence. Notably, hierarchical clustering has proven effective in identifying goat morphotypes based on morphometric traits, even when breeds and hybrids share the same geographical distribution [41]. In contrast, clustering based on morphometric indices failed to resolve distinct district-level groups, suggesting that raw linear body measurements provide superior discriminatory power in this context.

Interestingly, the Catacaos-dominated cluster consisted largely of animals belonging to four specific farmers, implicating husbandry practices as a potential driver of phenotypic differentiation alongside, or even beyond, geographic factors. This observation aligns with findings from Uganda, where agro-ecological management systems influenced morphological variation in indigenous goats [42], and with similar findings in other traditional production systems [34,43]. In Peru, considerable heterogeneity in production systems has been documented even within single regions [44–47], emphasizing the need to determine whether management strategies contribute more significantly than ecosystemic conditions to shaping goat morphology in Piura.

Principal component analysis (PCA) further clarified the structure of morphometric variation across districts. The first principal component (Dim1) explained 72.6% of the total variance, with positive contributions from all LBMs and body weight, representing a dominant body size-related axis. This strong unidimensional structure, reflecting a general size gradient from smaller to larger individuals, parallels patterns observed in other ruminants—for example, Sussex cattle, where Dim1 accounted for 61% of the variance [48]. Despite some overlap, district level differentiation along Dim1 was evident, with a distinct subset of Catacaos goats diverging from those in Chulucanas and Lancones. By comparison, Dim1 accounted for 21.1% of the variance in Tumbes goats, 43.18% in Chilean Creole goats, and 45.64% in Mexican Black Creole goats [12,32,49], highlighting the stronger dimensional consolidarion observed in our dataset. The second component (Dim2) showed low loading for BW but higher contributions from RH, WH, and CW, reflecting variation in body shape and proportionality rather than overall size.

Complementing the clustering and PCA results, trait-by-trait comparisons revealed that goats from Catacaos consistently exhibited larger values for all nine LBMs and five morphometric indices, defining a district-specific morphometric profile. As indicated by HCA, these differences likely reflect the influence of particular farming practices. By contrast, differences between Lancones and Chulucanas were limited to a smaller subset of traits. RF analysis identified BW, BD, and CP as the primary discriminators among districts, while the remaining LBMs exhibited lower yet meaningful discriminatory capacity. These same variables also showed high loadings on Dim1, reinforcing their central role in defining morphometric structure. While these traits may facilitate the identification of local morphotypes within Piura, their diagnostic utility may differ elsewhere, where ecological and management contexts diverge.

The directional trend toward larger body measurements observed in Catacaos goats—particularly in CG, CP, BD, and RW—is especially noteworthy, as it may indicate an orientation toward specific production types. While traits like CG and RW are critical for meat production and are known to have high heritability [30], others such as WH, which also increased in Catacaos goats, are typically associated with dairy-type animals. The simultaneous enhancement of vertical and horizontal body dimensions may therefore indicate a dual-purpose phenotype in Catacaos goats, potentially shaped by selection pressures favoring versatility in productive roles.

Morphometric indices provided additional resolution for evaluating inter-district variation. Across districts, Creole goats exhibited a broadly shared morphological pattern characterized by brevilinear body shape (BOI < 0.85), tall stature (PPI > 105), eumetric proportions (MPI ≈ 10–11), and relatively low compactness and pelvic development (CCI ≈ 260, TPI < 33, LPI < 37). This combination describes animals with pronounced thoracic development relative to body length—typical of meat-oriented phenotypes—alongside reduced body mass relative to height and short pelvic dimensions, traits more common in dairy types. Goats from Catacaos displayed significantly greater thoracic capacity, taller stature, stronger limbs, and higher compactness than their counterparts from Chulucanas and Lancones, but no distinct differences in pelvic dimensions. Collectively, these traits support the emergence of a dual-purpose morphotype in Catacaos, particularly among individuals within Cluster 2.

The morphometric divergence observed between Lancones and Catacaos mirrors trends reported elsewhere in Peru, where inter-district variation in LBMs has been noted [12,14]. Such distinctions reinforce the value of morphometric analysis for identifying local phenotypes, a method successfully applied to indigenous livestock in other countries [50]. Larger body dimensions in grazing ruminants are often associated with functional advantages: grater leg length facilitates longer foraging ranges [51], increased body depth enhances abdominal musculature [52], rumen capacity [53], and locomotive efficiency [54,55], while broader rump dimensions have been associated with improved reproductive performance [50]. Further research is required to determine whether these relationships hold true under the ecological and management conditions of Piura.

Geographic variation in ruminant morphology is widely documented, both within breeds [12] and across indigenous ecotypes [43]. Morphometric classifications have proven effective in delineating zones defined by vegetation and resource availability [56]. In our study, goats from Catacaos—an area with more consistent access to crop residues—exhibited larger body dimensions, consistent with previous findings where improved grazing or supplementation led to increased body size in goats [57]. Future research should incorporate quantitative assessments of diet and management to better disentangle environmental and genetic drivers of phenotypic variation.

This study represents one of the most comprehensive morphometric assessments of Peruvian Creole goats to date, underscoring the potential for localized phenotypic differentiation within the Piura region. However, caution is warranted when interpreting morphometric indices originally developed for cattle [30]; these metrics may require revalidation in goats, particularly in systems where animals exhibit dairy-like features but are primarily used for meat production. Importantly, morphometric variation alone may not fully capture the distinctiveness of regional goat types. Differences in management practices, crossbreeding, ecological context, and productivity can be equally defining. An integrated framework combining phenotypic, genetic, and production-system data is essential for accurately characterizing and conserving local goat populations. Such understanding not only supports biodiversity preservation but also underpins the valorization of native breeds through differentiated products and geographic indications—strategies that have proven effective in strengthening rural economies.

## 5. Conclusion

Our findings demonstrate clear morphometric differentiation among Creole goat populations within the Piura region, with LBMs outperforming morphometric indices in capturing district-level variation. The integral contribution of multiple LBMs underscores their utility in identifying meaningful phenotypic distinctions. Notably, a subgroup of larger-bodied goats in Catacaos exhibited marked divergence from those in Lancones, suggesting the presence of a distinct morphotype. While these districts occupy different SDF ecosystems, the underlying drivers—whether ecological, managerial, or nutritional—remain to be fully elucidated. Further research integrating environmental metrics, production system typologies, and forage availability is essential to clarify the mechanisms shaping morphological diversity in this region.

## Supporting information

S1 File Raw data. (XLSX)

S2 File Supporting information. (DOCX)
