# Supplementary material for: Creole goat morphological diversity partially mirrors district-level variation in the seasonally dry forest of Piura in Peru
Source: PLoS One. 2025 Dec 31;20(12):e0339584. doi: 10.1371/journal.pone.0339584 (PMC12755774; doi:10.1371/journal.pone.0339584)
Supplement: S2 File — (DOCX) [file pone.0339584.s002.docx]

**Supplementary material**

**“Caprine Morphological Diversity Partially Mirrors District-Level Variation in the Dry Seasonal Forest of Piura in Peru”**

**S Table 1.** **Correlation matrix for body weight and linear body measurement variables**

|  | **BW** | **WH** | **RH** | **BL** | **CG** | **CP** | **RL** | **BD** | **CW** | **RW** |
| --- | --- | --- | --- | --- | --- | --- | --- | --- | --- | --- |
| **BW** | 1.0000 | 0.6725 | 0.6996 | 0.7366 | 0.8739 | 0.7581 | 0.7210 | 0.7577 | 0.7138 | 0.7730 |
| **WH** | 0.6725 | 1.0000 | 0.8718 | 0.6693 | 0.6828 | 0.6652 | 0.6926 | 0.6992 | 0.5036 | 0.6586 |
| **RH** | 0.6996 | 0.8718 | 1.0000 | 0.6970 | 0.6854 | 0.6874 | 0.6942 | 0.6728 | 0.5361 | 0.6480 |
| **BL** | 0.7366 | 0.6693 | 0.6970 | 1.0000 | 0.7041 | 0.6846 | 0.6647 | 0.6741 | 0.4952 | 0.6921 |
| **CG** | 0.8739 | 0.6828 | 0.6854 | 0.7041 | 1.0000 | 0.7864 | 0.7446 | 0.8157 | 0.7294 | 0.7502 |
| **CP** | 0.7581 | 0.6652 | 0.6874 | 0.6846 | 0.7864 | 1.0000 | 0.6762 | 0.7554 | 0.6571 | 0.7067 |
| **RL** | 0.7210 | 0.6926 | 0.6942 | 0.6647 | 0.7446 | 0.6762 | 1.0000 | 0.6834 | 0.5406 | 0.7085 |
| **BD** | 0.7577 | 0.6992 | 0.6728 | 0.6741 | 0.8157 | 0.7554 | 0.6834 | 1.0000 | 0.6663 | 0.7068 |
| **CW** | 0.7138 | 0.5036 | 0.5361 | 0.4952 | 0.7294 | 0.6571 | 0.5406 | 0.6663 | 1.0000 | 0.6068 |
| **RW** | 0.7730 | 0.6586 | 0.6480 | 0.6921 | 0.7502 | 0.7067 | 0.7085 | 0.7068 | 0.6068 | 1.0000 |

**S Table 2.** **Correlation matrix for morphometric indices**

|  | **BOI** | **PVI** | **PPI** | **MTI** | **TPI** | **LPI** | **CCI** | **RCTI** | **CLI** |
| --- | --- | --- | --- | --- | --- | --- | --- | --- | --- |
| **BOI** | 1.0000 | -0.0119 | -0.4800 | 0.2977 | -0.0982 | -0.1441 | -0.2417 | -0.1656 | 0.1778 |
| **PVI** | -0.0119 | 1.0000 | -0.0852 | 0.0981 | 0.6633 | -0.3287 | 0.2508 | 0.1569 | -0.2026 |
| **PPI** | -0.4800 | -0.0852 | 1.0000 | -0.0135 | -0.4176 | -0.2038 | -0.3100 | -0.4603 | 0.1291 |
| **MTI** | 0.2977 | 0.0981 | -0.0135 | 1.0000 | 0.0589 | -0.0698 | 0.0115 | 0.5799 | 0.2745 |
| **TPI** | -0.0982 | 0.6633 | -0.4176 | 0.0589 | 1.0000 | 0.3573 | 0.5311 | 0.4801 | -0.3579 |
| **LPI** | -0.1441 | -0.3287 | -0.2038 | -0.0698 | 0.3573 | 1.0000 | 0.2647 | 0.2399 | -0.1690 |
| **CCI** | -0.2417 | 0.2508 | -0.3100 | 0.0115 | 0.5311 | 0.2647 | 1.0000 | 0.4633 | -0.8717 |
| **RCTI** | -0.1656 | 0.1569 | -0.4603 | 0.5799 | 0.4801 | 0.2399 | 0.4633 | 1.0000 | -0.0224 |
| **CLI** | 0.1778 | -0.2026 | 0.1291 | 0.2745 | -0.3579 | -0.1690 | -0.8717 | -0.0224 | 1.0000 |

**S Table 3. Number of goats per farmer assigned to HCA clusters**

| **District** | **Farmer** | **Cluster** | | | **Total** |
| --- | --- | --- | --- | --- | --- |
|  |  | **1** | **2** | **3** |  |
| Catacaos | **A** | 4 | 8 | 2 | **14** |
|  | **D** | 1 | 20 | 5 | **26** |
|  | **G** | 0 | 10 | 2 | **12** |
|  | **E** | 3 | 2 | 8 | **13** |
|  | **F** | 5 | 1 | 6 | **12** |
|  | **B** | 3 | 59 | 21 | **83** |
|  | **C** | 7 | 1 | 12 | **20** |
| Chulucanas | **M** | 9 | 0 | 1 | **10** |
|  | **R** | 5 | 0 | 3 | **8** |
|  | **V** | 7 | 0 | 6 | **13** |
|  | **O** | 8 | 1 | 3 | **12** |
|  | **D1** | 4 | 0 | 0 | **4** |
|  | **E1** | 1 | 0 | 2 | **3** |
|  | **X** | 20 | 3 | 5 | **28** |
|  | **A1** | 7 | 1 | 1 | **9** |
|  | **S** | 6 | 0 | 1 | **7** |
|  | **W** | 2 | 2 | 5 | **9** |
|  | **Y** | 1 | 0 | 5 | **6** |
|  | **T** | 33 | 3 | 31 | **67** |
|  | **B1** | 1 | 2 | 10 | **13** |
|  | **U** | 8 | 0 | 5 | **13** |
|  | **N** | 15 | 0 | 0 | **15** |
|  | **P** | 15 | 0 | 3 | **18** |
|  | **Q** | 30 | 0 | 7 | **37** |
|  | **C1** | 2 | 0 | 2 | **4** |
|  | **Z** | 15 | 0 | 8 | **23** |
| Lancones | **I** | 12 | 0 | 3 | **15** |
|  | **H** | 9 | 0 | 3 | **12** |
|  | **L** | 11 | 0 | 1 | **12** |
|  | **K** | 49 | 1 | 27 | **77** |
|  | **J** | 15 | 0 | 7 | **22** |
|  | **Total** | **308** | **114** | **195** | **617** |

**S Table 4.** **Distribution (%) of goats per farmer assigned to HCA clusters**

| **District** | **Farmer** | **Cluster** | | | **Total** |
| --- | --- | --- | --- | --- | --- |
|  |  | **1** | **2** | **3** |  |
| Catacaos | **A** | 28.6 | 57.1 | 14.3 | **100** |
|  | **D** | 3.8 | 76.9 | 19.2 | **100** |
|  | **G** | 0.0 | 83.3 | 16.7 | **100** |
|  | **E** | 23.1 | 15.4 | 61.5 | **100** |
|  | **F** | 41.7 | 8.3 | 50.0 | **100** |
|  | **B** | 3.6 | 71.1 | 25.3 | **100** |
|  | **C** | 35.0 | 5.0 | 60.0 | **100** |
| Chulucanas | **M** | 90.0 | 0.0 | 10.0 | **100** |
|  | **R** | 62.5 | 0.0 | 37.5 | **100** |
|  | **V** | 53.8 | 0.0 | 46.2 | **100** |
|  | **O** | 66.7 | 8.3 | 25.0 | **100** |
|  | **D1** | 100.0 | 0.0 | 0.0 | **100** |
|  | **E1** | 33.3 | 0.0 | 66.7 | **100** |
|  | **X** | 71.4 | 10.7 | 17.9 | **100** |
|  | **A1** | 77.8 | 11.1 | 11.1 | **100** |
|  | **S** | 85.7 | 0.0 | 14.3 | **100** |
|  | **W** | 22.2 | 22.2 | 55.6 | **100** |
|  | **Y** | 16.7 | 0.0 | 83.3 | **100** |
|  | **T** | 49.3 | 4.5 | 46.3 | **100** |
|  | **B1** | 7.7 | 15.4 | 76.9 | **100** |
|  | **U** | 61.5 | 0.0 | 38.5 | **100** |
|  | **N** | 100.0 | 0.0 | 0.0 | **100** |
|  | **P** | 83.3 | 0.0 | 16.7 | **100** |
|  | **Q** | 81.1 | 0.0 | 18.9 | **100** |
|  | **C1** | 50.0 | 0.0 | 50.0 | **100** |
|  | **Z** | 65.2 | 0.0 | 34.8 | **100** |
| Lancones | **I** | 80.0 | 0.0 | 20.0 | **100** |
|  | **H** | 75.0 | 0.0 | 25.0 | **100** |
|  | **L** | 91.7 | 0.0 | 8.3 | **100** |
|  | **K** | 63.6 | 1.3 | 35.1 | **100** |
|  | **J** | 68.2 | 0.0 | 31.8 | **100** |

Orange color indicates farmers from Catacaos which contributed more than 50% of their animals to cluster 2. Blue indicates the same for Lancones farmers in Cluster 1.

**S Table 5.** **Loadings of LBMs to the principal components**

| **Morphometric traits** | **Dim.1** | **Dim.2** | **Dim.3** | **Dim.4** | **Dim.5** |
| --- | --- | --- | --- | --- | --- |
| **BW** | 0.823 | 0.025 | 0.009 | 0.001 | 0.014 |
| **WH** | 0.698 | 0.165 | 0.070 | 0.000 | 0.001 |
| **RH** | 0.713 | 0.145 | 0.068 | 0.001 | 0.012 |
| **BL** | 0.680 | 0.036 | 0.101 | 0.113 | 0.011 |
| **CG** | 0.837 | 0.038 | 0.001 | 0.001 | 0.006 |
| **CP** | 0.752 | 0.011 | 0.000 | 0.023 | 0.065 |
| **RL** | 0.701 | 0.018 | 0.026 | 0.182 | 0.010 |
| **BD** | 0.764 | 0.012 | 0.004 | 0.004 | 0.093 |
| **CW** | 0.568 | 0.253 | 0.089 | 0.000 | 0.045 |
| **RW** | 0.726 | 0.002 | 0.065 | 0.017 | 0.040 |

BW: body weight; WH: withers height; RH: rump height; BL: body length; CG: chest girth; CP: cannon perimeter; RL: rump length; BD: body depth; CW: chest width, RW: rump width

**S Table 6.** **Loadings of the morphometric indices to the principal components**

| **Morphometric Indices** | **Dim.1** | **Dim.2** | **Dim.3** | **Dim.4** | **Dim.5** |
| --- | --- | --- | --- | --- | --- |
| **BOI** | 0.051 | 0.444 | 0.058 | 0.297 | 0.056 |
| **PVI** | 0.198 | 0.119 | 0.487 | 0.094 | 0.076 |
| **PPI** | 0.187 | 0.255 | 0.005 | 0.438 | 0.002 |
| **MTI** | 0.009 | 0.468 | 0.080 | 0.191 | 0.162 |
| **TPI** | 0.689 | 0.040 | 0.019 | 0.000 | 0.188 |
| **LPI** | 0.096 | 0.086 | 0.487 | 0.072 | 0.080 |
| **CCI** | 0.748 | 0.104 | 0.002 | 0.000 | 0.136 |
| **RCTI** | 0.411 | 0.157 | 0.264 | 0.070 | 0.005 |
| **CLI** | 0.357 | 0.321 | 0.111 | 0.031 | 0.156 |

BOI: body index; PVI: pelvic index; PPI: proportionality index; MTI: metacarpal-thoracic index; TPI: transversal pelvic index; LPI: longitudinal pelvic index; CCI; compactness index; RCTI: relative cannon thickness index; CLI: cannon load index.

**S Table 7a. PERMANOVA table of body weight and linear body measurements under reduced model**

| **Source of variation** | **DF** | **Sum of Sqs** | **R2** | **F** | ***p*-value** |
| --- | --- | --- | --- | --- | --- |
| **District** | 2 | 41041 | 0.24571 | 126.5343 | 0.001 |
| **Age** | 3 | 26395 | 0.15803 | 54.2533 | 0.001 |
| **District:Age** | 6 | 1478 | 0.00885 | 1.5187 | 0.130 |
| **Residual** | 605 | 98115 | 0.58741 |  |  |
| **Total** | 616 | 167029 | 1.00000 |  |  |

**S Table 7b. PERMANOVA table of morphometric indices under reduced model**

| **Source of variation** | **DF** | **Sum of Sqs** | **R2** | **F** | ***p*-value** |
| --- | --- | --- | --- | --- | --- |
| **District** | 2 | 12718 | 0.06786 | 26.1603 | 0.001 |
| **Age** | 3 | 25137 | 0.13412 | 34.4686 | 0.001 |
| **District:Age** | 6 | 2490 | 0.01329 | 1.7072 | 0.038 |
| **Residual** | 605 | 147067 | 0.78473 |  |  |
| **Total** | 616 | 187412 | 1.00000 |  |  |

**S Table 8. Mean decrease in the Gini coefficients for body weight and LBMs**

| **Variable** | **MeanDecreaseGini** |
| --- | --- |
| BD | 60.07163 |
| BW | 59.40819 |
| CP | 50.78043 |
| RH | 38.06384 |
| CW | 36.53485 |
| CG | 34.30125 |
| WH | 33.48741 |
| RL | 27.20820 |
| BL | 26.62631 |
| RW | 20.76500 |

BW: body weight; WH: withers height; RH: rump height; BL: body length; CG: chest girth; CP: cannon perimeter; RL: rump length; BD: body depth; CW: chest width, RW: rump width

**S Fig 1. Out-of-bag error plot of Random Forest model for district classification of goats by morphometric traits.**


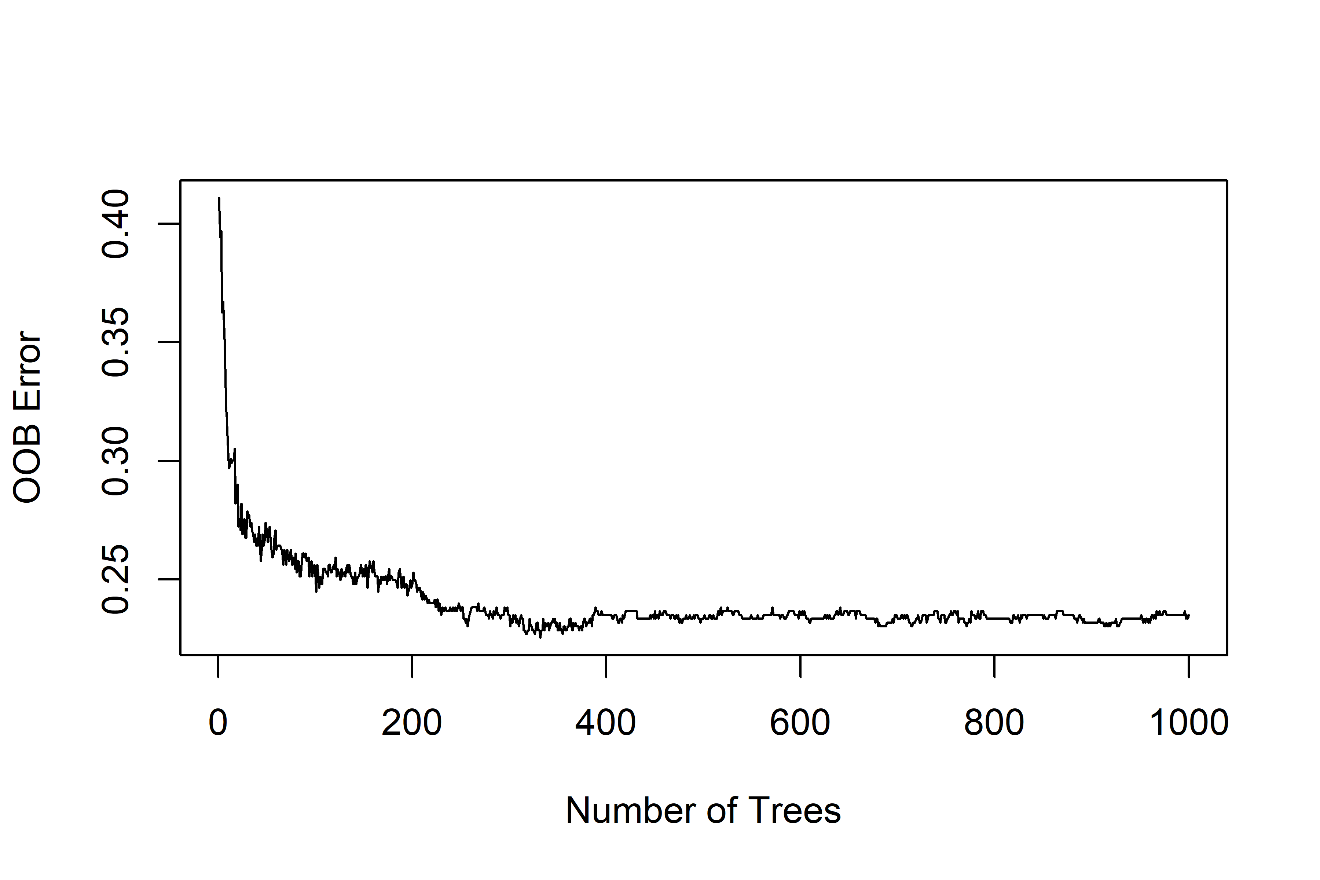


Curve stability is considered at 400 trees, to be used in the RF analysis.
